# Cone-Beam Computerized Tomography Evaluation of the Relationship between Orthodontic Vertical Direction Parameters and the Distance from the Apex of the Upper Central Tooth to the Nasal Floor and Anterior Nasal Spine

**DOI:** 10.3390/tomography10010004

**Published:** 2024-01-05

**Authors:** Saadet Çınarsoy Ciğerim, Türkan Sezen Erhamza

**Affiliations:** 1 Department of Orthodontics, Faculty of Dentistry, Van Yuzuncu Yil University, 65090 Van, Turkey; 2 Department of Orthodontics, Faculty of Dentistry, Kirikkale University, 71450 Kırıkkale, Turkey; turkansezen@kku.edu.tr

**Keywords:** orthodontic treatment, upper central, anterior nasal spine, nasal floor, CBCT, vertical growth pattern

## Abstract

The aim of this study was to examine the relationship between the vertical cephalometric values and the distance from the apex tip of the upper central tooth (U1A) to the anterior nasal spine (ANS) and nasal floor (NF) using cone-beam computerized tomography (CBCT). One hundred and twenty-two patients who applied to the Department of Orthodontics between January 2011 and June 2019 were included. The distances between the U1A and the NF and ANS were measured using CBCT. Statistical significance was considered as *p* < 0.05. Of the 122 individuals, 73.8% (n = 90) were female and 26.2% (n = 32) were male, with a mean age of 22.8 ± 3.3 years. A statistically significant moderate positive correlation was found between the mean NF-U1A values and the N-Me, ANS-Me, ANS-Gn, S-Go, and N-ANS measurements (*p* < 0.01). A statistically significant positive correlation was found between the mean ANS-U1A values and the Ar-Go-Me, total posterior angles, N-Me, SN/GoGn and Y-axis angle, ANS-Me, and ANS-Gn measurements (*p* < 0.01). The distance from the U1A to the ANS and NF was related to the orthodontic vertical direction parameters. The ANS-U1A and NF-U1A distances can serve as reference points for identifying the orthodontic vertical growth pattern from CBCT scans.

## 1. Introduction

Cephalometric analysis is the most common diagnostic method for patients undergoing orthodontic treatment. Cephalometric analysis can evaluate the vertical, sagittal, and transversal relationships of the upper and lower jaw with respect to the skull base; the relationship between the upper and lower jaw; and the positions of the teeth with respect to the relevant base and each other [1,2,3]. Angle measurements have been determined by applying various planes to radiographs for many years. Broadbent proposed a facial growth model in 1937 [4] before Brodie introduced an evaluation method for the growth pattern of the head, brain, nose, dental arches, and lower jaw [5]. Later, Björk evaluated the cranial base and its growth, concluding that the vertical enlargement of the face is closely related to the growth rotation of the lower jaw [2].

The measurement of the sella–nasion/gonion–gnathion (SN/GoGn) angle is commonly used to determine the orthogonal morphology and growth pattern of the face [3]. McNamara suggested using the gonion–menton/Frankfurt horizontal plane (GoMe/FH) angle for vertical growth patterns [6]. Matilla et al. suggested considering the gonial angle independent of the cranial base in orthopantomograms [7]. The Y-axis angle can also be used in the classification of vertical facial anomalies [8]. A study on the distribution of the lower jaw rotation model according to the sagittal jaw position found that the latter was independent of the vertical facial growth model and lower jaw rotation [6]. Evaluations using SN/GoGn, GoMe/FH, and gonial angle norms provided conflicting information for individuals with Angle’s Class II and Class III deviations [9].

The anterior nasal spine (ANS) is a fixed reference that is not affected by orthodontic tooth movements, and it is one of the cephalometric landmarks in the skull [10]. The A-point is the deepest point of the anterior alveolar bone recess below the ANS. Since the A-point is on the alveolar bone, it is affected by tooth movements [8]. The nasion (N) is the depression at the root of the nose corresponding to the nasofrontal suture [11]. The nasal floor (NF) and ANS are fixed reference points that do not change during orthodontic treatment.

The use of cone-beam computed tomography (CBCT) is becoming widespread in orthodontics as in all areas of dentistry. The main reason for this is that (CBCT) gives the chance of a three-dimensional examination in the evaluation of hard tissues in the jaws. In addition, large-volume CBCT images eliminate the need for extra panoramic, cephalometric, and other 2D radiographs [12]. CBCT is mostly used in orthodontics to evaluate impacted, ectopic, and transposed teeth. In addition, CBCT is used in dentofacial–orofacial deformities and anomalies, facial asymmetry, cleft lip and palate, TMJ disorders, oral region pathologies, and airway evaluation, which are closely related to orthodontics [13,14,15,16,17,18,19]. The routine use of cbct in orthodontics is generally not recommended. The main reason for this is the radiation dose to which the patient is exposed. The majority of orthodontic patients are children, and they are more sensitive to radiation [20]. Nowadays, with the developing technology, the radiation dose of newly produced CBCTs is decreasing compared to their predecessors, but it is still high compared to 2D radiographs such as panoramic and cephalometric [21]. In addition, the cost of CBCT images is higher than that of 2D radiographs [22]. The probability of incidental findings in individuals undergoing CBCT for orthodontic purposes is around 69.5%. Two-dimensional images are inadequate, especially in the evaluation of the nasal cavity and maxillary sinus. It is known that the use of CBCT in orthodontics has many advantages. In particular, it is obvious that coincidental findings that may be missed in 2D radiographs can be recognized in CBCT, and this will contribute positively to the diagnosis and treatment planning in orthodontics [23].

Vertical malocclusions are some of the most challenging cases to treat for orthodontists. Therefore, information on the vertical alignment of the face is very important in terms of diagnosis, treatment planning, and prognosis [24]. Although only a few studies have calculated the distances between the apex of the upper central incisor (U1A) and other anatomical structures, the literature suggests that these distances are related to sagittal or vertical direction parameters. Therefore, we hypothesized that the distance from the U1A to the NF and ANS might be related to the vertical direction parameters. The aim of this study was to examine the relationship between the vertical cephalometric values and the distance from the U1A to the ANS and NF using cone-beam computed tomography (CBCT) and to evaluate whether this distance could be applied as a parameter for determining the vertical skeletal growth pattern.

## 2. Materials and Methods

Our cross-sectional retrospective study included patients who applied to Van Yüzüncü Yıl University, Faculty of Dentistry, Department of Orthodontics, between January 2011 and June 2019. After planning the study, approval was obtained from the Ethics Committee of Van Yüzüncü Yıl University for Non-Interventional Clinical Research (decision no. 2019/06-04). Patients’ demographic information and medical and dental anamnesis were obtained from the Orthodontic Clinic records before orthodontic treatment. The study was conducted in accordance with the ethical principles of the Helsinki Declaration. We considered skeletal and Angle class 1 (ANB: 0–4°) patients with upper inclination (UI-NA distance: 0–4 mm) and a normal upper incisor-NA angle (UI-NA angle: 22 ± 3°) and without an open bite before orthodontic treatment. Patients aged 18 years and older who were systemically healthy according to ASA classification (ASA1) were included. Patients with dental anomalies such as root resorption, dental invagination, taurodontism, or dilation in their upper central incisors before treatment; a cleft lip or palate; low-quality radiographs; or previous orthodontic treatment were excluded.

### 2.1. Cephalometric Measurements

All cephalometric X-rays were taken on the same device by the same operator, with the lips in the resting position and the head in the natural position. We obtained lateral cephalometric radiographs with a Sirona Orthophos XG imaging system under standard conditions. Hard and soft tissue points and measurements were determined using NemoCeph V.2017. Lateral cephalometric X-rays taken at the beginning of the treatment were examined and evaluated by a single observer (S.Ç.C.), and the measurements were repeated on the same radiographs after 4 weeks.

**Figure 1:**
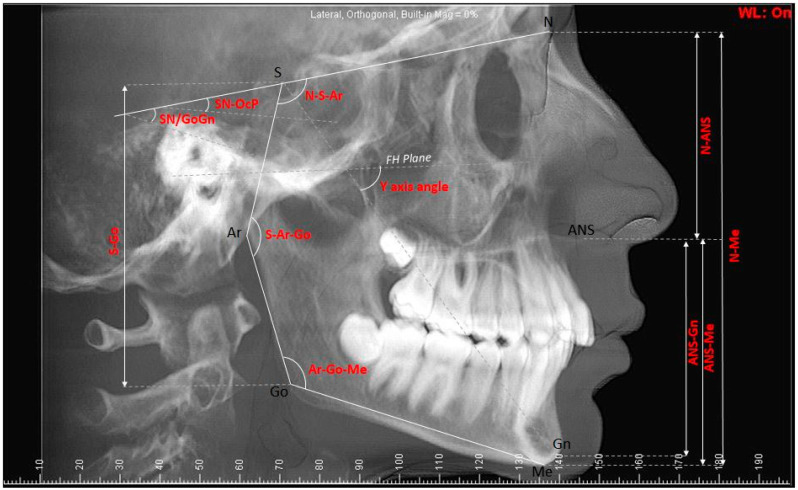
Cephalometric measurements used in the study: The saddle angle (N-S-AR), articular angle (S-Ar-Go), gonial angle (Ar-Go-Me), anterior facial height (N-Me), posterior facial height (S-Go), sella–nasion/gonion–gnathion (SN/GoGn), nasion–anterior nasal spine (N-ANS), anterior nasal spine–menton (ANS-Me), anterior nasal spine–gnathion (ANS-Gn), sella–gonion (S-Go), Y-axis angle, and sella nasion–occlusal plane (SN-OcP).

The vertical cephalometric measurements used in the study were the saddle angle (N-S-AR), articular angle (S-Ar-Go), gonial angle (Ar-Go-Me), sum of posterior angles, anterior facial height (N-Me), posterior facial height (S-Go), sella–nasion/gonion–gnathion (SN/GoGn), nasion–anterior nasal spine (N-ANS), anterior nasal spine–menton (ANS-Me), anterior nasal spine–gnathion (ANS-Gn), sella–gonion (S-Go), Y-axis angle, and sella nasion–occlusal plane (SN-OcP) (Figure 1).

### 2.2. CBCT Measurements

**Figure 2:**
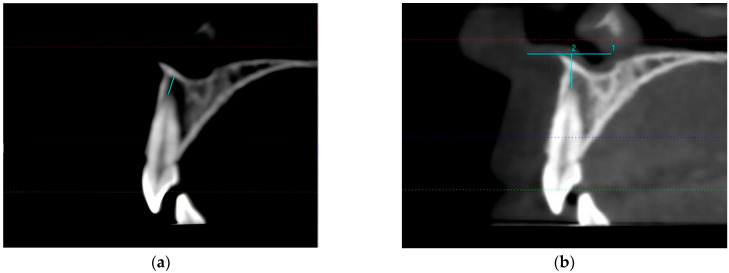
(**a**) Measurements from UA1 to NF were performed parallel to the long axis of the tooth in the cross-sectional sagittal view. (**b**) The vertical distance of the horizontal line passing through the ANS to the upper central apical point was taken as a reference when measuring the distance between the U1A and ANS in the cross-sectional sagittal view.

Individuals subjected to CBCT (120 kV, 130 fov, 0.300 voxel 5 mA; KaVo 3D eXam (Biberach, Germany)) for various orthodontic reasons were included. Measurements were made by examining the CBCT images taken before treatment. All evaluations were carried out by the same investigator (S.Ç.C.), and radiographs were measured again after 4 weeks. ExamVision was used to measure the distance between the U1A and ANS and NF on the CBCT images. The NF-UA1 distance was measured from the closest point of the apex tip of the upper central tooth to the NF for standardization, and we ensured that the measurement line was parallel to the long axis of the tooth. The vertical distance of the horizontal line passing through the ANS to the apex tip of the upper central tooth was taken as a reference when measuring the distance between the U1A and ANS (Figure 2).

The right NF-U1A (NF to apex tip of upper-right central tooth), left NF-U1A (NB to apex tip of upper-left central tooth), right ANS-U1A (ANS to apex tip of upper-right central tooth), and left ANS-U1A (ANS to apex tip of upper-left central tooth) distances were measured using CBCT. The NF-U1A and ANS-U1A values were obtained by calculating the mean of the right- and left-side measurements, respectively. Regarding the ANS-Me and ANS-Gn distances and Y-axis angles, the patients were divided into 3 groups in terms of growth status. For the ANS-Me distance, 63.9 mm and below was considered decreased, 64 mm to 74.9 mm normal, and 75 mm and above increased. For the ANS-Gn distance, 63.9 mm and below was considered decreased, 64 mm to 72.9 mm normal, and 73 mm and above increased. For the Y-axis angle, 52.9 degrees and below was considered decreased, 53 degrees to 66.9 degrees normal, and 67 degrees and above increased.

### 2.3. Statistical Analysis

For the statistical analysis of the study data, NCSS (Number Cruncher Statistical System) 2007 (Kaysville, UT, USA) and descriptive statistical methods (mean, standard deviation, median, frequency, ratio, minimum, and maximum) were used. The conformity of the quantitative data to a normal distribution was tested with the Kolmogorov–Smirnov and Shapiro–Wilk tests and graphical evaluations. Pearson correlation analysis was used to evaluate the relationships between variables showing normal distribution. Student’s *t*-test was used to compare two groups of quantitative data showing normal distribution. The Kruskal–Wallis test was used for comparisons of three or more groups that did not show normal distribution, and the Bonferroni–Dunn test was used for pairwise comparisons. The level of significance was accepted as *p* < 0.05. The correlation coefficient (r) was evaluated according to the following criteria: 0–0.25, very weak; 0.26–0.49, poor; 0.50–0.69, medium; 0.70–0.89, good; 0.90–1.00, very good. Post hoc power measurements were above 80% for all significant results.

The sample size was calculated using G*Power (version 3.1.7) software, with a minimum requirement of 111 individuals. The study considered 122 patients who met the inclusion criteria during the relevant time frame, meeting the minimum sample size requirements.

## 3. Results

The ages of the 122 individuals included in the study ranged from 18 to 30, with a mean age of 22.8 ± 3.3 years. Of these individuals, 73.8% (n = 90) were female and 26.2% (n = 32) were male. The intra-examiner correlation coefficient indicated high reliability between the two measurements (for CBCT measurements, r = 0.92; for cephalometric radiography measurements, r = 0.91).

### 3.1. Relationship between NF-U1A and Cephalometric Measurements

We found no statistically significant relationship between NF-U1A and NS-AR, S-Ar-Go, Ar-Go-Me, sum of posterior angles, SN/GoGn, and SN-OcP measurements (*p* > 0.05). A statistically significant moderate positive correlation was found between NF-U1A and N-Me, ANS-Me, and ANS-Gn measurements (r = 0.547, r = 0.585, r = 0.611, respectively; *p* < 0.01). A statistically significant weak positive correlation was found between NF-U1A and S-Go and N-ANS measurements (r = 0.372 and r = 0.338, respectively; *p* < 0.01). A statistically significant very weak positive correlation was found between NF-U1A and Y-axis angle measurements (r = 0.219; *p* < 0.05). See Table 1 for details.

### 3.2. Relationship between ANS-U1A and Cephalometric Measurements

A statistically significant weak positive correlation was found between ANS-U1A and Ar-Go-Me, sum of posterior angles, N-Me, SN/GoGn, and Y-axis angle measurements (r = 0.287, r = 0.311, r = 0.384, r = 0.297, r = 0.311, respectively; *p* < 0.01). A statistically significant moderate positive correlation was found between ANS-U1A and ANS-Me and ANS-Gn measurements (r = 0.534 and r = 0.531, respectively; *p* < 0.01). See Table 1 for details.

The mean NF and ANS measurement values of the patients according to gender did not show a statistically significant difference (*p* > 0.05), (Table 2).

The distribution of ANS-Me, ANS-Gn, and Y-axis angle values is shown in Table 3.

### 3.3. Evaluation of ANS-U1A and NF-U1A Measurements According to ANS-Me Value

A statistically significant difference was found between NF-U1A measurements according to the ANS-Me level (*p* = 0.001; *p* < 0.01). Based on the paired comparisons, the NF-U1A measurements of the groups with increased and normal ANS-Me values were higher than those of the decreased group (*p* = 0.001 and *p* = 0.001, respectively; *p* < 0.01). No statistically significant difference was found between the NF-U1A measurements of the groups with increased and normal ANS-Me values (*p* > 0.05). A statistically significant difference was found between ANS-U1A measurements according to the ANS-Me level (*p* = 0.001; *p* < 0.01). Based on the paired comparisons, the ANS-U1A measurements of the groups with increased and normal ANS-Me values were higher than those of the decreased group (*p* = 0.002 and *p* = 0.001, respectively; *p* < 0.01). No statistically significant difference was found between the ANS-U1A measurements of the groups with increased and normal ANS-Me values (*p* > 0.05). See Table 4 for details.

### 3.4. Evaluation of ANS-U1A and NF-U1A Measurements According to ANS-Gn Value

A statistically significant difference was found between NF-U1A measurements according to the ANS-Gn value (*p* = 0.001; *p* < 0.01). Based on the paired comparisons, the NF-U1A measurements of the groups with increased and normal ANS-Gn values were higher than those of the decreased group (*p* = 0.001 and *p* = 0.001, respectively; *p* < 0.01). No statistically significant difference was found between the NF-U1A measurements of the groups with increased and normal ANS-Gn values (*p* > 0.05). A statistically significant difference was found between ANS-U1A measurements according to the ANS-Gn value (*p* = 0.001; *p* < 0.01). Based on the paired comparisons, the ANS-U1A measurements of the groups with increased and normal ANS-Gn values were higher than those of the decreased group (*p* = 0.001 and *p* = 0.001, respectively; *p* < 0.01). No statistically significant difference was found between the ANS-U1A measurements of the groups with increased and normal ANS-Gn values (*p* > 0.05). See Table 5 for details.

## 4. Discussion

The normal values for cephalometric analyses are generally considered to differ according to ethnicity [25,26,27]. Some normal values for dimensional measurements used in Tweed’s analysis do not fit the Turkish ethnicity [28]. Gazilerli redesigned Steiner’s analysis for Turkish children aged 13–16 [29]. Işımer et al. [30] found that the normal gonial angle in Turkish differed by nearly 10 degrees from Björk’s measurements [31]. For these reasons, racial characteristics should also be considered while performing cephalometric evaluations.

Tsunori et al. reported that facial types, in terms of morphological characteristics, are an important factor to be considered in orthodontic treatment because they influence the prediction of the maxillofacial growth within the anchorage system used during treatment [32]. Ligthelm-Bakker et al. found a negative correlation between the growth rates of the upper and lower anterior facial height, suggesting that some children show accelerated growth in the lower compared to the upper facial height, and vice versa [33]. Janson et al. confirmed that the anterior ratio can be used in orthodontic diagnosis in addition to numerical vertical measurements [34].

Maxillary anterior teeth are important for not only dental and facial aesthetics but also physiological functions such as pronunciation and chewing [35,36,37,38,39]. Therefore, determining the three-dimensional (3D) position of the maxillary incisors is integral to orthodontic diagnosis, and various biomechanical treatment plans and methods are used to achieve the ideal incisor position. Vertical malocclusions are very challenging, with treatment often failing to achieve the desired aesthetic results and the possibility of post-treatment relapse. Therefore, information on the vertical alignment of the face is very important for diagnosis, treatment planning, and prognosis [24]. Although only a few studies have calculated the distances between the U1A and other anatomical structures, the literature suggests that these distances are related to sagittal or vertical direction parameters. Therefore, we hypothesized that the distance from the U1A to the NF and ANS might be related to the vertical direction parameters.

Cho et al. reported that the anteroposterior distance between the maxillary central incisor roots and the incisive canal is approximately 5–6 mm. Evaluating the proximity of the incisive canal to the maxillary incisors, in addition to its dimensions, may be helpful in cases where a significant amount of maxillary retraction is planned [40].

A study examining the correlation coefficients of GoGn/SN, gonial angle, and GoMe/FH measurements for the vertical classification of the face reported that these three angles support each other in revealing the vertical positions in individuals with hyperdivergent facial structures [41]. We observed that as the distance from the U1A to the ANS and NF increased, the ANS-Gn, ANS-Me, and N-Me distances and Y-axis angle increased, showing a correlation with each other.

Gracco et al. reported that for the upper incisors, the face type is statistically significantly associated with both the thickness of the alveolar bone and the distance between the root apex and the lingual cortex [42]. We observed that the vertical evaluation parameters (N-Me, ANS-Me, ANS-Gn, and Y-axis angle measurements) were positively correlated with the distance from the U1A to the ANS and NF.

Sadek et al. examined alveolar bone thickness and height using CBCT and reported that patients with larger vertical dimensions showed increased anterior dentoalveolar height in both the maxilla and mandible without a significant difference in posterior alveolar height [43]. We also evaluated patients with different vertical dimensions. Similarly to Sadek et al., we observed that the distance from the U1A to the ANS and NF increased in patients with an increased lower anterior facial height.

Enoki et al. examined the dental–skeletal dimensions and variations in the lower facial height in individuals during the growth and development period [44]. Similarly to Enoki et al., we observed that the distance from the U1A to the ANS and NF increased in patients with an increased lower anterior face height. Additionally, we observed that as the distance between the U1A and NF increased, the N-Me, ANS-Me, ANS-Gn, S-Go, and N-ANS distances and Y-axis angle increased. Furthermore, as the distance between the U1A and ANS increased, the ANS-Gn, ANS-Me, and N-Me distances; Ar-Go-Me, SN/GoGn, and Y-axis angles; and the sum of posterior angles increased.

The ANS-Me distance is the most commonly used measurement in orthodontics for the assessment of vertical direction and defines the height of the lower anterior face [45]. Unlike other measurements in the vertical direction, the ANS-Me distance is a linear distance measurement and is frequently used as a reference in studies [46]. The reason for comparing ANS-Me and ANS-Gn distances with UA1-ANS and UA1-NF distances in this study is that ANS-Me and ANS-Gn distances are linear distance measurements and commonly used and accepted vertical direction reference parameters. In addition, the teeth are located in the lower anterior region, and we thought that ANS-Me and ANS-Gn would be the parameters that would be least affected by possible errors and deviations in the comparison of UA1-NF and UA1-ANS measurements in this region. Deoghare et al. used the ANS-Gn distance to evaluate the lower anterior face height in their study and showed that this distance varies according to different types of malocclusions [47]. In this study, only skeletal and Angle class 1 patients were included to eliminate the effect of this factor. There is no study in the literature where the ANS-Me or ANS-Gn distance is compared with UA1. In addition, no study was found in the literature that used the UA1-ANS and UA1-NF distance as a reference and compared this distance with ANS-Me and ANS-Gn. Since there is no similar study, it was not possible to compare our results.

The limitations of this study included the small number of patients with increased growth in the group comparisons performed according to the ANS-Me and ANS-Gn growth direction model during the statistical evaluations. In addition, the Y-axis angle groups could not be compared in terms of NF-U1A and ANS-U1A because the differences between the groups formed for the Y-axis angle, which is another growth direction parameter, were not statistically significant.

## 5. Conclusions

The present study was the first to use the UA1-NF and UA1-ANS distances as references. These distances were found to be correlated with the vertical direction parameters ANS-Me and ANS-Gn. It was revealed that UA1-NF and UA1-ANS distances were higher in individuals with normal lower anterior face height than in individuals with reduced lower anterior face height. These results support that UA1-NF and UA1-ANS distances can be used as reference parameters in the assessment of vertical direction in orthodontics. Further studies are needed to support the use of UA1-NF and UA1-ANS distances as reference distance measurements.

## Figures and Tables

**Table 1:** Relationships between cephalometric measurements and NF-U1A and ANS-U1A.

Vertical Direction Parameters	NF-U1A r *p*	ANS-U1A r *p*
N−S−AR	−0.065 0.476	0.017 0.853
S−Ar−Go	0.030 0.740	−0.068 0.458
Ar−Go−Me	0.099 0.278	0.287 0.001 **
Sum of posterior angles	0.098 0.284	0.311 0.001 **
N−Me	0.547 0.001 **	0.384 0.001 **
S−Go	0.372 0.001 **	0.131 0.150
SN/GoGn	0.064 0.484	0.297 0.001 **
N−ANS	0.338 0.001 **	0.079 0.388
ANS−Me	0.585 0.001 **	0.534 0.001 **
ANS−Gn	0.611 0.001 **	0.531 0.001 **
Y−axis angle	0.219 0.015 *	0.311 0.001 **
SN−OcP	−0.163 0.073	0.082 0.369

r: Pearson correlation coefficient; ** *p* < 0.01; * *p* < 0.05.

**Table 2:** Evaluation of the mean NF-UA1 and ANS-UA1 measurement values according to gender.

		Female (n = 90)	Male (n = 32)	*p*
NF-UA1 distance (mm)	Mean ± Sd	5.57 ± 2.37	6.14 ± 2.27	^a^ 0.243
	Min–Max	1.1–11.4	2.1–11.1	
ANS-UA1 distance (mm)	Mean ± Sd	5.93 ± 2.27	5.62 ± 2.53	^a^ 0.516
	Min–Max	1–11	1.5–12.2	

^a^ Student’s *t*-test, Sd: standard deviation.

**Table 3:** Distribution of ANS-Me, ANS-Gn, and Y-axis angle values.

	Level	n	%
ANS-Me	Decreased (≤63.9)	66	54.1
	Normal (64–74.9)	49	40.2
	Increased (≥75)	7	5.7
ANS-Gn	Decreased (≤63.9)	76	62.3
	Normal (64–72.9)	36	29.5
	Increased (≥73)	10	8.2
Y-Axis Angle	Decreased (≤52.9)	3	2.4
	Normal (53–66.9)	100	82
	Increased (≥67)	19	15.6

**Table 4:** Evaluation of ANS-U1A and NF-U1A measurements according to ANS-Me values.

ANS-Me Level		NF-U1A	ANS-U1A
Decreased (≤63.9)	n	66	66
	Min–max	1.1–10.1	1.5–10
	Mn ± SD	4.57 ± 1.84	4.86 ± 1.91
Normal (64–74.9)	n	49	49
	Min–max	2.2–11.4	1–11
	Mn ± SD	6.94 ± 2.13	6.80 ± 2.15
Increased (≥75)	n	7	7
	Min–max (median)	5.5–10.8	5–12.2
	Mn ± SD	8.06 ± 2.35	8.54 ± 2.70
	^a^ *p*	0.001 **	0.001 **

^a^ Kruskal–Wallis test; ** *p* < 0.01.

**Table 5:** Evaluation of ANS-U1A and NF-U1A measurements according to ANS-Gn values.

ANS-Gn Level		NF-U1A	ANS-U1A
Decreased (≤63.9)	n	76	76
	Min–max	1.1–9.3	1.5–10
	Mn ± SD	4.76 ± 1.88	5.01 ± 1.88
Norm (64–72.9)	n	36	36
	Min–max	2.2–11.4	1–11
	Mn ± SD	7.03 ± 2.19	7.00 ± 2.36
Increased (≥73)	n	10	10
	Min–Max	5.5–10.8	5–12.2
	Mn ± Sd	8.30 ± 2.08	8.11 ± 2.33
	^a^ *p*	0.001 **	0.001 **

^a^ Kruskal–Wallis test; ** *p* < 0.01.

## Data Availability

The data resulting from this study are available from the authors.
